# Hydrolytic function of Exo1 in mammalian mismatch repair

**DOI:** 10.1093/nar/gku420

**Published:** 2014-05-14

**Authors:** Hongbing Shao, Celia Baitinger, Erik J. Soderblom, Vickers Burdett, Paul Modrich

**Affiliations:** 1 Department of Biochemistry, Duke University Medical Center, Durham, NC 27710, USA; 2 Howard Hughes Medical Institute, Duke University Medical Center, Durham, NC 27710, USA; 3 Proteomics Core Facility, Duke University Medical Center, Durham, NC 27710, USA

## Abstract

Genetic and biochemical studies have previously implicated exonuclease 1 (Exo1) in yeast and mammalian mismatch repair, with results suggesting that function of the protein in the reaction depends on both its hydrolytic activity and its ability to interact with other components of the repair system. However, recent analysis of an Exo1-E109K knockin mouse has concluded that Exo1 function in mammalian mismatch repair is restricted to a structural role, a conclusion based on a prior report that N-terminal His-tagged Exo1-E109K is hydrolytically defective. Because Glu-109 is distant from the nuclease hydrolytic center, we have compared the activity of untagged full-length Exo1-E109K with that of wild type Exo1 and the hydrolytically defective active site mutant Exo1-D173A. We show that the activity of Exo1-E109K is comparable to that of wild type enzyme in a conventional exonuclease assay and that in contrast to a D173A active site mutant, Exo1-E109K is fully functional in mismatch-provoked excision and repair. We conclude that the catalytic function of Exo1 is required for its participation in mismatch repair. We also consider the other phenotypes of the Exo1-E109K mouse in the context of Exo1 hydrolytic function.

## INTRODUCTION

Exonuclease 1 (Exo1), a 5′ to 3′ hydrolytic activity of the Rad2 family, has been implicated in multiple genetic stabilization pathways including mismatch repair, double-strand break repair and telomere maintenance (reviewed in (1)). The enzyme was discovered as a *Schizosaccharomyces pombe* activity whose level increases during meiosis (2). The finding that inactivation of the *S. pombe EXO1* gene leads to mitotic mutability and increased recombinant yield in intragenic meiotic crosses suggested that Exo1 may function during mismatch repair (3), an idea supported by the finding that *S. pombe msh2Δ* and *exo1Δ* are epistatic in an *ade6–51* reversion assay (4).

Yeast and human Exo1 have been shown to interact with MSH2, MSH3 and MLH1, subunits of the mismatch repair initiating activities MutSα (MSH2·MSH6), MutSβ (MSH2·MSH3) and MutLα (MLH1·PMS2 in mammals, MLH1·PMS1 in yeast) (5–8). Analysis of extract reactions and biochemical reconstitution experiments have directly implicated Exo1 in mammalian mismatch repair, with the latter experiments indicating involvement of Exo1 hydrolysis during the excision step of repair (9–13). Two reconstituted systems that support mismatch-provoked excision have been described. The simplest, in which excision is directed by a strand break 5′ to the mispair, depends on MutSα, MutLα, Exo1 and RPA (replication protein A) (11–13). In this reaction, MutSα activates Exo1 and renders the exonuclease highly processive (11,12). RPA controls processive action of the MutSα·Exo1 complex, resulting in attenuation of hydrolysis upon mismatch removal. MutLα is not required for excision in this system but acts together with MutSα to modestly suppress hydrolysis on mismatch-free DNA, and the mismatch-dependence of excision can be further enhanced by the presence of PARP-1 (poly[ADPribose] polymerase 1) (14).

A second mode of mismatch-provoked excision that supports both 5′- and 3′-heteroduplexes depends on activation of the MutLα endonuclease in a reaction that requires a mismatch, MutSα (or MutSβ), RFC (replication factor C) and PCNA (proliferating cell nuclear antigen) (15,16). The orientation of PCNA loading onto the helix confers strand directionality on MutLα incision. This restricts endonuclease action to one strand and serves to bracket the mismatch with 3′ and 5′ breaks. 5′-termini produced in this manner serve as entry sites for MutSα-activated Exo1, which removes the mismatch by a 5′ to 3′ hydrolytic mechanism presumed to be similar to that described above (15).

Genetic studies with yeast (reviewed in (1)) and mice (17) have shown that Exo1 deficiency confers only a partial defect in mismatch repair. Biochemical studies have yielded similar conclusions, demonstrating that Exo1-deficient mouse cell extracts retain significant residual mismatch repair activity (17,18), indicating existence of one or more Exo1-independent modes of mismatch repair. One possible mechanism for such events has been suggested by biochemical experiments, which have shown that a mismatch can be removed from a MutLα-incised heteroduplex by an Exo1-independent reaction that relies on synthesis-driven strand displacement by DNA polymerase δ (18).

Heterozygotic germline defects in genes encoding MSH2, MLH1, MSH6 or PMS2 have been identified as a cause of hereditary non-polyposis colorectal cancer (HNPCC) (19). A screen for Exo1 defects in 225 suspected HNPCC patients led to identification of 13 heterozygotic germline missense mutations (20). Two of these, E109K and L410R, were reported to be defective in exonuclease activity but otherwise structurally stable as judged by analysis of N-terminal His-tagged derivatives (21). However, and contrary to expectation, tumors in 12 of the 13 putative HNPCC patients were found to have lost the mutant Exo1 allele, retaining the wild type copy of the gene (20). In fact, a link between Exo1 mutations and HNPCC has been questioned in subsequent studies, which have shown that a number of the Exo1 missense mutations identified by Wu *et al.* (20) are common variants in the human population (22) and that individuals with heterozygotic germline Exo1 deletions apparently do not develop HNPCC (23).

Yeast genetic experiments have suggested that Exo1 participation in mismatch repair *in vivo* depends on both its hydrolytic activity (24–26) and its ability to interact with other components of the repair system (8,24,27). By contrast, a recent study of mice homozygous for the Exo1-E109K mutation mentioned above has concluded that Exo1 involvement in mammalian mismatch repair is restricted to a structural function (28). This conclusion is based on the finding that the mutant protein is hydrolytically defective (21) or nearly so (28), and the fact that unlike Exo1 null mice, the mutability of animals homozygous for the Exo1-E109K mutation is similar to that of wild type mice (28). Because this conclusion is difficult to reconcile with the repair studies described above and because E109 is distant from the Exo1 active site (29), we have examined the activities of the untagged form of Exo1-E109K. We show that in contrast to previous reports (21,28), Exo1-E109K is as active as wild type enzyme in conventional exonuclease assay and is fully functional in mismatch-provoked excision and repair *in vitro*. However, a D173A active site mutant that retains native exonuclease domain structure (29) is hydrolytically deficient and repair defective.

## MATERIALS AND METHODS

### Proteins and extracts

The Exo1-E109K mutant was constructed by PCR-based mutagenesis of the pFastbBac1 Exo1 plasmid (10), changing codon 109 from GAG to AAG. The coding sequence of the mutant gene was established by sequencing it in its entirety and further confirmed as described below. Recombinant human MutSα (9,30), MutLα (30), DNA polymerase δ (18), Exo1 (10), Exo1-D173A (9) and Exo1-E109K were isolated as described previously from SF9 cells infected with appropriate baculovirus expression vectors. Recombinant human RPA and PCNA were purified from *E. coli* (9,11,14,31). Human RFC was isolated from HeLa nuclear extract (9,32). Proteins were diluted into 10 mM Hepes-KOH, pH 7.6, 1 mM dithiothreitol, 200 mM KCl, 2 mg/ml BSA, 10% glycerol. Whole cell extracts were prepared from immortalized *Mlh1****^−/−^*** *Exo1****^−/−^*** mouse embryo fibroblast (MEF) cells according to Kadyrov *et al.* (18).

Presence of the appropriate amino acids at positions 109 and 173 of Exo1, Exo1-E109K and Exo1-D173A was confirmed in two ways. Sheared DNA was prepared from the lysates of baculovirus-infected SF9 cells used for protein isolation. A segment extending from the polyhedrin promoter through the Exo1 Y341 codon was PCR amplified using primers d(GATTATTCATACCGTCCCACCAT) and d(GTAGTCATCGATCTGTTCAAAAG), and the sequence of the PCR product determined.

In the second approach, isolated proteins were analyzed by nano-flow liquid chromatography electrospray ionization tandem mass spectrometry (LC-MS/MS). Purified Exo1, Exo1-E109K and Exo1-D173A were buffer exchanged into 50 mM ammonium bicarbonate (pH 8) using a ZebaSpin (Pierce) gel filtration column and then supplemented with 1% Rapigest (Waters) surfactant. Samples were reduced with 10 mM dithiothreitol for 15 min at 70°C, alkylated with 25 mM iodoacetamide for 30 min at room temperature and digested with chymotrypsin (Roche) for 18 h at 37°C. Following acidification of samples with 0.1% trifluoroacetic acid to hydrolyze the Rapigest surfactant, samples were lyophilized and stored at −80°C until the LC-MS analysis was performed. Lyophilized peptides were resuspended in 12 μl of 2% acetonitrile, 0.1% formic acid prior to LC-MS/MS analysis. Chromatographic separation was performed on a Waters NanoAcquity UPLC equipped with a 1.7 μm BEH130 C_18_ 75 μm I.D. × 250 mm reversed-phase column. The mobile phase consisted of (A) 0.1% formic acid in water and (B) 0.1% formic acid in acetonitrile. Following a 5 μl injection, peptides were trapped for 5 min on a 5 μm Symmetry C_18_ 180 μm I.D. × 20 mm column at 5 μl/min in 99.9% A. The analytical column was then switched in-line and a linear elution gradient of 5% B to 40% B was performed over 90 min at 300 nl/min. The analytical column was connected to a fused silica PicoTip emitter (New Objective, Cambridge, MA, USA) with a 10 μm tip orifice and coupled to a Waters Synapt G2 QToF mass spectrometer through an electrospray interface. The instrument was operated in data-dependent mode of acquisition with precursor MS scans from *m/z* 50–2000 and the top three most abundant precursor ions being subjected to MS/MS fragmentation. For all experiments, charge-dependent collisionally-induced dissociation energy settings were employed and a 120 s dynamic exclusion was employed for previously fragmented precursor ions.

LC-MS/MS data files were processed in Mascot distiller (Matrix Science) and then submitted to independent Mascot database searches (Matrix Science) against a SwissProt (taxonomy *Homo sapiens*) database (20353 forward sequences, updated November 2013) appended with the reverse sequence of all of the forward entries. Search tolerances were 5 ppm for precursor ions and 0.04 Da for product ions using chymotrypsin specificity with up to three missed cleavages. Carbamidomethylation (+57.0214 Da on C) was set as a fixed modification, whereas oxidation (+15.9949 Da on M), Asp to Ala modification (−43.9898 Da on D) and Glu to Lys (−0.9476 Da on E) were considered as variable modifications. All searched spectra were imported into Scaffold (Proteome Software) and protein confidence thresholds were set using a Bayesian statistical algorithm based on the PeptideProphet and ProteinProphet algorithms which yielded a peptide and protein false discovery rate of 0% (33,34). The annotated Scaffold file (Shao_et_al_Exonuclease.sf3) has been uploaded to the Duke Institute for Genome Science and Policy's express data repository: https://discovery.genome.duke.edu/express/resources/3629/Shao_et_al_Exonuclease.sf3. To estimate peptide isoform abundances within a sample, raw LC-MS data was imported into Skyline (MacCoss Laboratory, University of Washington) and full MS extracted ion chromatograms (EICs) were performed for each qualitatively identified peptide using retention time and isotope distribution to assign correct peak.

### Exonuclease 1 assay

Conventional exonuclease assay was scored on a synthetic 5′-recessed oligonucleotide duplex (35). Synthetic d(pGGATCCCCGCTAGCGGGTACCGAGCTCGAATTCACTGG) was ^32^P-labeled by the polynucleotide kinase exchange reaction (36), hybridized to unphosphorylated d(CCAGTGAATTCGAGCTCGGTACCCGCTAGCGGGGATCCTCTA) and the labeled duplex purified by chromatography on a Waters GenPak Fax column. Exo1 activity was determined at 37°C in a buffer similar to that used for mismatch-provoked excision assay. Reactions (25 μl) contained 20 mM Tris-HCl pH 7.6, 0.75 mM HEPES-KOH, 120 mM KCl, 250 μg/ml bovine serum albumin, 1.5 mM adenosine triphosphate (ATP), 1 mM glutathione, 5 mM MgCl_2_, 1% glycerol, 0.06 mM dithiothreitol and ^32^P-labeled oligonucleotide duplex and Exo1 as indicated. Five-μl samples were withdrawn at intervals and added to 5 μl of 90% formamide, 10 mM ethylenediaminetetraacetic acid (EDTA), 0.05% bromophenol blue and 0.05% xylene cyanol. Samples were heated to 90°C for 5 min, and release of [^32^P]dGMP scored by thin-layer chromatography on PEI-cellulose plates (Merck KGaA) in 0.1 M phosphate buffer pH 7.0. Radioactivity was quantitated using a phosphorimager. Steady-state kinetic parameters were determined at enzyme concentrations of 0.1–0.2 nM.

### Mismatch repair and mismatch-provoked excision assays

Mismatch repair and mismatch-provoked excision was scored using circular 6440 bp f1MR G-T heteroduplexes that contained a strand break 128 bp 5′ (as viewed along the shorter path in the circular substrate) or 141 bp 3′ to the mismatch (9) by minor modifications of published procedures (9,11,37). 5′-directed excision in a 4-protein system (11) was determined in 20 μl reactions containing 25 mM Tris-HCl, pH 7.5, 4 mM HEPES -KOH pH 7.5, 125 mM KCl, 1.5 mM ATP, 100 ng 5′-heteroduplex, 1 mM reduced glutathione, 0.4 mM dithiothreitol, 0.8 mg/ml bovine serum albumin, 4% glycerol and 100 ng MutSα, 50 ng MutLα, 150 ng RPA and 4 ng wild type or mutant Exo1 as indicated. 5′- and 3′-directed excision in the 6-protein system (9) was determined in a similar manner except reactions also contained 66 ng RFC, 25 ng PCNA and 100 ng 5′- or 3′-heteroduplex as indicated. After incubation at 37°C for 8 min, reactions were terminated by addition of 30 μl of 8 mM Tris-HCl, pH 8.0, 25 mM EDTA, 0.2% SDS, 0.2 mg/ml glycogen and 1 mg/ml proteinase K. After proteinase K digestion, phenol extraction and ethanol precipitation, recovered DNA was digested with ClaI and NheI to score gapped DNA (9).

5′- and 3′-directed mismatch repair using purified components was performed as in the 6-protein excision system except that the reactions also contained 200 μM dNTPs and 20 ng recombinant polymerase δ, and incubation was for 5 min (5′-heteroduplex) or 20 min (3′-heteroduplex). Reactions were quenched, DNA was isolated as described above and correction of the G-T mismatch was determined after cleavage with HindIII and ClaI (37). Mismatch repair in the presence of 60 μg of *Mlh1****^−/−^*** *Exo1****^−/−^*** MEF cell extract was performed in similar manner except that ATP, bovine serum albumin and glycerol concentrations were 3 mM, 0.1 mg/ml and 0.5%, respectively, and the 20 μl reactions contained 50 ng 5′- or 3′-heteroduplex DNA. Extracts were supplemented with 70 ng MutLα and 5 ng Exo1 variants, as indicated. Incubation was at 37°C for 30 min. Reactions were quenched and repair determined as described above.

## RESULTS

### Wild type and mutant Exo1 polypeptides

The Exo1-E109K mutation was identified in several putative HNPCC families (20). Based on comparison of N-terminal His-tagged wild type and E109K proteins, Sun *et al.* (21) concluded that residue Glu-109 is required for Exo1 hydrolytic activity. We have revisited this conclusion for several reasons. Glu-109, which is not conserved among Exo1 homologs (38), is located far from the hydrolytic center of the enzyme (29). Furthermore, because the Exo1 N-terminus resides within the active site (29), presence of a N-terminal His-tag might interfere with hydrolytic function.

**Figure 1.**
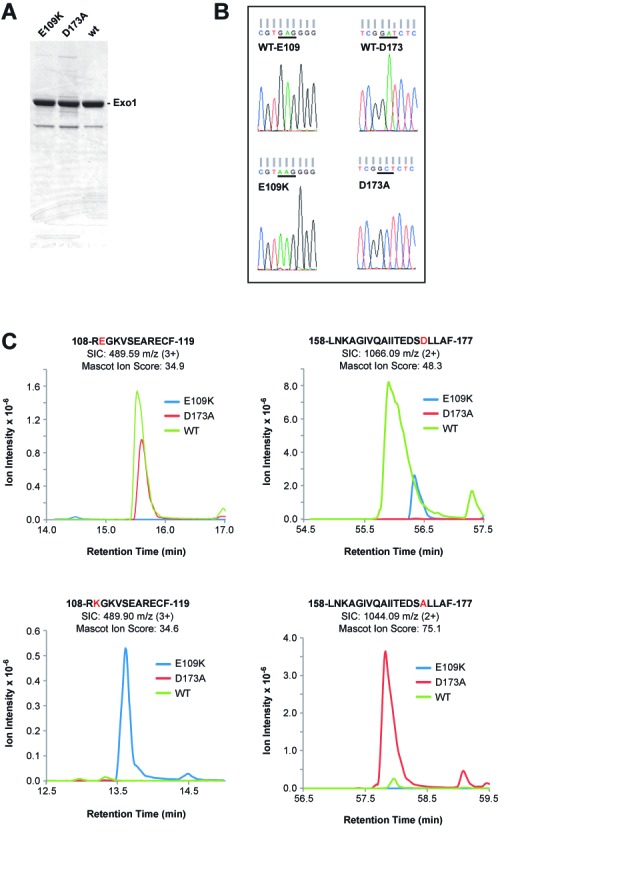
Purity and sequence confirmation of Exo1 and Exo1 variants used in this study. (A) Samples (5 μg) of Exo1 preparations used in this study were subjected to polyacrylamide gel electrophoresis in the presence of sodium dodecyl sulfate and stained with Coomassie Brilliant Blue. (B) Lysates of baculovirus-infected SF9 cells employed for protein isolation were used as sources of template DNA for PCR amplification of the N-terminal 341 codons of Exo1. DNA sequence analysis of PCR products demonstrated presence of Glu and Asp codons at positions 109 and 173 for wild type Exo1 (upper reads); Lys at position 109 for Exo1-E109K (lower left read) and Ala at position 173 for Exo1-D173A (lower right read). (C) EIC peak traces of chymotryptic products spanning residues E109 and D173 across three LC-MS injections. Each EIC was performed ±20 ppm around the monoisotopic precursor *m*/*z*. Peaks were manually verified using retention time relative to qualitative peptide identification time as well as predicted ratio of C12 and C13 isotopomer peaks.

Untagged, full-length (846 residues) wild type Exo1, Exo1-E109K and Exo1-D173A were extensively purified from SF9 cells infected with the corresponding baculovirus expression vectors. The three proteins fractionated in the same manner, and isolated preparations had an estimated purity of 90% (Figure 1A). Exo1-D173A, an active site mutant, which retains native conformation of the exonuclease domain (9,24,29,39), was used as a negative control for hydrolytic function. Sequences of wild type and mutant polypeptides were validated in two ways. Sheared DNA was prepared from the lysates of baculovirus-infected SF9 cells used for protein isolation. PCR amplification of a segment extending from the polyhedrin promoter through the Exo1 Y341 codon followed by sequence analysis of isolated PCR products demonstrated the presence of appropriate Lys or Ala codons in Exo1-E109K and Exo1-D173A, respectively (Figure 1B). Presence of the correct amino acids within the vicinity of residues 109 and 173 was further confirmed by LC-MS analysis of chymotrypsin digests of the three purified proteins. As shown in Figure 1C, the peptide _108_R**E**GKVSEARECF_119_ was recovered only from wild type and Exo1-D173A digests, while _108_R**K**GKVSEARECF_119_ was obtained from the digest of Exo1-E109K. Similarly, _158_LNKAGIVQAIITEDS**D**LLAF_177_ was identified only in wild type and Exo1-E109K digests, while _158_LNKAGIVQAIITEDS**A**LLAF_177_ was recovered from the chymotrypsin digest of Exo1-D173A.

### Exo1-E109K is hydrolytically functional in conventional exonuclease assay

**Figure 2.**
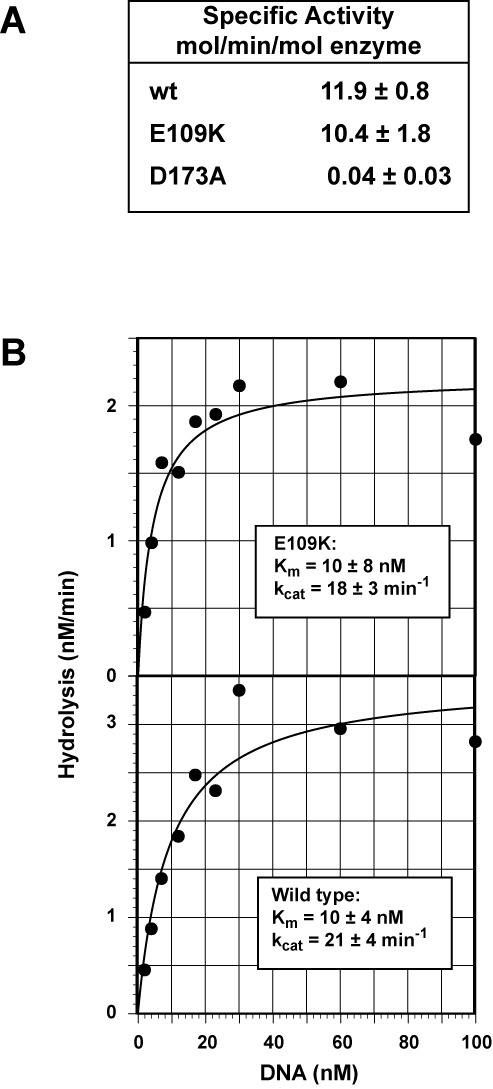
Exo1-E109K is a functional exonuclease. (A) Activities of wild type, Exo1-E109K and Exo1-D173A were determined as a function of enzyme concentration (0.01–2 nM) using a synthetic 5′-recessed ^32^P-labeled oligonucleotide duplex (27.5 nM, Materials and Methods). Specific activities shown were determined from progress curves where rates were linear with enzyme concentration. Results shown are the mean of 3 (wild type and Exo1-D173A) or 4 (Exo1-E109K) determinations (±one standard deviation). (B) Steady-state rates of [^32^P] synthetic duplex hydrolysis by 0.15 nM wild type Exo1 or Exo1-E109K were determined as a function of substrate concentration and results fit to a hyperbola using the non-linear regression function of DeltaGraph (RedRock Software). K_m_ and k_cat_ values shown in the insets are averages of three independent determinations (±one standard deviation).

A previous analysis of the Exo1 hydrolytic domain has demonstrated that the enzyme displays similar activities on blunt, 5′-recessed and nicked DNA duplexes (35). We therefore compared the activity of untagged full-length Exo1-E109K with that of wild type Exo1 and hydrolytically defective Exo1-D173A using a 5′-^32^P-recessed synthetic duplex substrate. As shown in Figure 2A, the activity of Exo1-E109K on this substrate is similar to that of wild type enzyme, but the hydrolytic activity of Exo1-D173A is less than 1% of that of the former two proteins. Steady-state kinetic analysis of hydrolysis occurring on the recessed duplex confirmed that the hydrolytic proficiencies of wild type Exo1 and Exo1-E109K are similar. As shown in the insets in Figure 2B, K_m_ and k_cat_ values for the two enzymes do not differ significantly.

It is noteworthy that the specific activity values reported here for untagged wild type enzyme and Exo1-E109K (Figure 2A) are several orders of magnitude higher than those observed by Sun *et al.* (21) with N-terminal His-tagged wild type Exo1. The k_cat_ values we have determined for untagged full-length Exo1 and Exo1-E109K are also 10- to 100-fold higher than those reported by Schaetzlein *et al.* (28) for His-tagged variants of the murine C-terminal truncated Exo1 hydrolytic domain.

### Exo1-E109K is functional in mismatch repair in mouse cell extracts but Exo1-D173A is not

**Figure 3.**
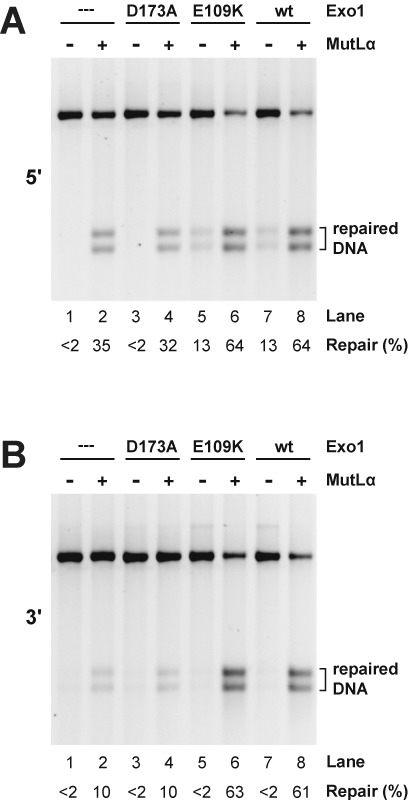
The exonuclease function is required for Exo1-dependent mismatch repair in mouse cell extract. Mismatch repair was scored using G-T heteroduplex DNA containing a 5′- (panel A) or 3′- (panel B) strand break in whole cell extract prepared from *Mlh1****^−/−^****Exo1****^−/−^*** MEF cells. Extracts were supplemented with MutLα as indicated and assays performed in the absence (dilution buffer only) or presence of Exo1-D173A, Exo1-E109K or wild type Exo1 (Materials and Methods). Mismatch repair, which converts the heteroduplex to a HindIII-sensitive form, was determined after cleavage with HindIII and ClaI.

To determine whether Exo1-E109K supports mismatch repair in an extract system, we utilized whole cell extracts derived from immortalized *Mlh1****^−/−^*** *Exo1****^−/−^*** MEF cells (18). The *EXO1* gene in these cells contains an internal deletion that results in loss of codons 181–252 (17) and elements of the catalytic domain (29). Extracts of these MLH1- and Exo1-deficient cells are devoid of mismatch repair activity on circular 6.4 kB G-T heterodouplex DNAs that contain a strand break 128 bp 5′ or 141 bp 3′ to the mismatch (lane 1 of Figure 3A and B). Extract supplementation with only MutLα restored significant levels of repair on both 5′- and 3′-heteroduplex DNAs (compare lanes 1 and 2, panels A and B), confirming previous findings that mouse cell extracts support significant levels of Exo1-independent mismatch repair (17,18).

Supplementation with wild type Exo1 alone also restored modest levels of repair on the 5′-heteroduplex (Figure 3A, lanes 1 and 7), an effect that was not observed with the 3′-substrate (Figure 3B, lanes 1 and 7), consistent with previous observations that a significant component of 5′-directed mismatch repair can occur in a MutLα-independent manner (10,13,37,40,41). Addition of Exo1-E109K to the MLH1- and Exo1-deficient extract resulted in repair comparable to that obtained with the wild type exonuclease (Figure 3A, lanes 5 and 7), but hydrolytically defective Exo1-D173A was inert in this regard, yielding background levels similar to the buffer control (compare lane 3 with lanes 1, 5 and 7).

Supplementation of the repair-defective extract with both MutLα and wild type Exo1, or MutLα and Exo1-E109K resulted in robust enhancement of both 5′- and 3′-directed mismatch repair. By contrast, the repair efficiency observed upon supplementation with MutLα and Exo1-D173A is indistinguishable from that observed in the presence of MutLα alone (Figure 3A and B, compare lanes 2, 4, 6 and 8). Because the D173A active site mutant retains native exonuclease domain structure (29), we conclude that hydrolytic functionality is required for the Exo1-dependent component of mismatch repair that occurs in mouse cell extracts.

### Exo1-E109K is functional in reconstituted mismatch-provoked excision

Several purified systems have been described that support Exo1-dependent mismatch-provoked excision *in vitro*. The simplest of these depends on four proteins (MutSα, MutLα, Exo1, RPA) and supports excision directed by a 5′-strand break (11–13). Excision in this system relies on mismatch-dependent activation of Exo1 by MutSα. MutLα and RPA are not essential for Exo1 activation in this manner but play regulatory roles in controlling Exo1 action. The second system, which supports excision directed by either a 5′- or 3′-strand break requires PCNA and RFC in addition to these 4 proteins (9). Excision by this 6-protein system depends on mismatch-, MutSα-, RFC- and PCNA-dependent activation of the MutLα endonuclease, which brackets the mismatch with 5′- and 3′-strand breaks (15). 5′-strand breaks produced in this manner are postulated to serve as entry sites for MutSα-activated Exo1, which removes the mismatch.

**Figure 4.**
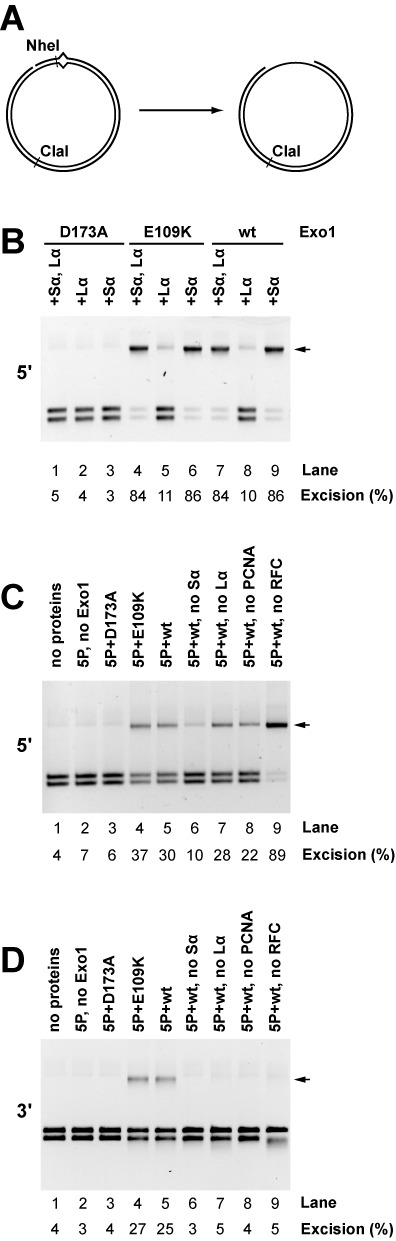
Mismatch-provoked excision in reconstituted systems requires the Exo1 hydrolytic function. (A) G-T heteroduplexes used in this study contain a NheI site that is located 5 bp distal to the mismatch in the 5′-heteroduplex and 5 bp proximal to the mispair in the 3′-heteroduplex. Mismatch provoked excision, which renders this region single-stranded and NheI-resistant (9,10), was scored by cleavage with NheI and ClaI. Arrows designate excision products in panels B–D*.* (B) 5′-directed excision on the G-T heteroduplex was determined in the 4-protein system. Reactions contained RPA with MutSα and MutLα present as indicated. Excision was scored in the presence of Exo1-D173A, Exo1-E109K or wild type enzyme (Materials and Methods). (C) 5′-directed excision in the 6-protein system was determined in reactions containing MutSα, MutLα, RPA, RFC, PCNA (indicated as 5P in lane labels), in the absence (lane 2) or presence of Exo1-D173A, Exo1-E109K or wild type Exo1 (lanes 3–5). Lanes 6–9 correspond to reactions containing wild type Exo1 with omissions as indicated. (D) Reactions were as in panel C except the heteroduplex strand break was located 3′ to the mismatch.

Mismatch-provoked excision can be monitored in several ways (9,11,42), but the simplest assay scores conversion of a restriction site located a short distance from the mismatch to an endonuclease-resistant form (Figure 4A). Figure 4B compares wild type Exo1, Exo1-E109K and Exo1-D173A with respect to the ability to support 5′-directed excision in the 4-protein system as scored by this method. As expected, excision requires hydrolytically functional Exo1 and MutSα, and is independent of the presence of MutLα (lanes 1–3 and 7–9). Exo1-E109K is as effective as wild type Exo1 in supporting this reaction (compare lanes 4–6 with 7–9).

As observed previously (9), the requirements for 5′-directed mismatch-provoked excision in the 6-component system are similar to those of the simpler system. Excision depends strongly on the presence of MutSα and Exo1 (Figure 4C, lanes 2, 5 and 6), but omission of MutLα or PCNA has little effect on product yield (lanes 7 and 8). Omission of RFC results in increased excision (lanes 5 and 8). Suppression of 5′ to 3′ excision by RFC, which has been observed previously, depends on the ligase homology domain of the large subunit of the clamp loader (9), an effect that may be due to the affinity of this domain for 5′-phosphoryl termini (43). As observed for the 5′-directed reaction in the 4-component system, Exo1-E109K is as active as the wild type enzyme in supporting excision in the 6-protein system (Figure 4C, lanes 4 and 5), but Exo1-D173A is non*-*functional (lane 3).

In contrast to 5′-directed excision in the 6-component system, excision directed by a 3′-strand break requires Exo1, MutSα, MutLα, PCNA and RFC (9), and these requirements are recapitulated in Figure 4D (lanes 2 and 5–9). Exo1-E109K is also functional in this reaction (lane 4), but Exo1-D173A is not (lane 3). RFC function in the control of 5′ to 3′ Exo1 hydrolysis from the heteroduplex 3′-strand break (9) is also evident in this figure. The small NheI-ClaI restriction fragment derived from the 3′-heteroduplex (Figure 4A) contains the 3′-strand break that directs excision. Non-specific 5′ to 3′ Exo1 hydrolysis from this break leads to degradation of this fragment (lane 9), an effect that is largely suppressed by the presence of RFC (lanes 2–8).

### Exo1-E109K is functional in reconstituted mismatch repair

**Figure 5.**
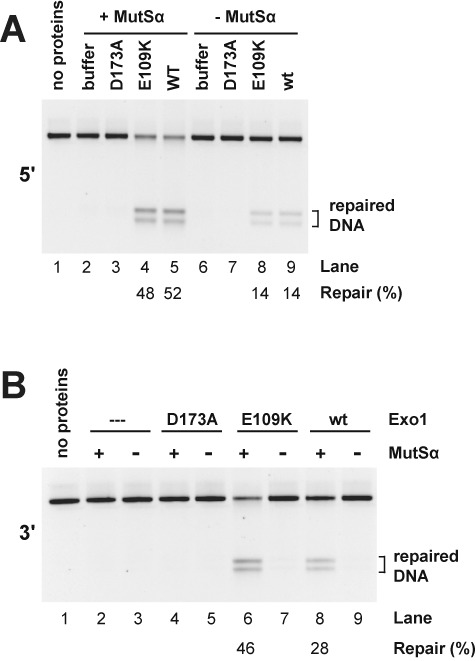
Exo1 hydrolytic action is required for reconstituted mismatch repair. Mismatch repair of 5′- (panel A) or 3′- (panel B) G-T heteroduplex DNAs was determined in the presence of MutLα, RPA, RFC, PCNA and DNA polymerase δ in the absence or presence of MutSα, Exo1-D173A, Exo1-E109K or wild type Exo1 as indicated. Mismatch repair was determined as in Figure 3.

Supplementation of MutSα, MutLα, Exo1, RPA, PCNA and RFC, the components required for bidirectional excision, with DNA polymerase δ and the dNTPs yields a minimal system that supports both 5′- and 3′-directed mismatch repair (37). As shown in Figure 5, wild type Exo1 and Exo1-E109K support MutSα-dependent mismatch correction on 5′- (panel A, compare lanes 4 and 5 with 8 and 9) or 3′-heteroduplex DNA (panel B, compare lanes 6 and 7, and lanes 8 and 9). However, as observed in cell extracts, hydrolytically deficient Exo1-D173A is incapable of supporting the repair of either heteroduplex in this purified system (panel A, lane 3; panel B, lane 4).

## DISCUSSION

Genetic studies in yeast have indicated both structural (8,27) and catalytic (24–26) functions for Exo1 in mismatch repair. Biochemical experiments utilizing mammalian cell lysates or partially fractionated extracts have also implicated Exo1 in a substantial component of MutSα- and MutLα-dependent mismatch repair events (10,17,18). These extract studies did not address the issue of structural versus catalytic involvement of the enzyme in mammalian repair, but analysis of several reconstituted human mismatch repair systems has demonstrated that the hydrolytic function of Exo1 plays an important role in the excision step of the reaction (9,11,13). The recent report that involvement of mouse Exo1 in mismatch repair is restricted to a structural role (28) was therefore surprising.

As discussed above, the interpretation of this mouse study is based on the premise that the Exo1-E109K mutant polypeptide is structurally stable but hydrolytically defective (28), a conclusion based on assay of His-tagged full-length (21) or C-terminal-truncated (28) Exo1 preparations that were expressed in *E. coli* and purified by Ni^2+^-chelation chromatography. Exo1-E109K isolates obtained in this manner had greatly reduced activity relative to wild type controls (21,28), but reported activities for parallel wild type control preparations are 1–3 orders of magnitude less than that found by others for the isolated hydrolytic domain (29,35) or that reported here for untagged full-length Exo1 and Exo1-E109K (Figure 2). The reduced activity of these His-tagged isolates, which could reflect presence of the tag or the nature of the C-terminal truncation, coupled with the use of a single-column isolation procedure may have rendered comparative analysis of the wild type and E109K proteins subject to interference by trace contamination with other nucleases.

Because the Exo1-D173A active site mutant retains native exonuclease domain structure (29), this mutant protein would presumably support structural functions of the polypeptide in mismatch repair. In fact, Exo1-D173A has been shown to be inactive in a reconstituted mismatch-provoked excision system where it also inhibits excision supported by the wild type enzyme (9). We have confirmed and extended these findings with the demonstration that the D173A variant also fails to support mismatch repair in cell extracts and in a reconstituted system (Figures 3 and 5). Exo1 hydrolytic action is therefore required for its function in mismatch repair *in vitro*, and presumably *in vivo* as well.

The results described here may have additional implications concerning the phenotypes of mice homozygous for the Exo1-E109K mutation (*Exo1^EK/EK^*). In addition to their mismatch repair-proficient phenotype, *Exo1^EK/EK^* mice differ from *Exo1^null/null^* animals in several other respects. Although *Exo1^null/null^* animals are defective in meiosis, and in the class switch recombination and somatic hypermutation steps of immunoglobulin gene maturation, these processes occur normally in *Exo1^EK/EK^* mice (28). Functionality of Exo1-E109K in these pathways has led to the conclusion that Exo1 involvement in each case is restricted to a structural role, but wild type behavior in these contexts may well be due to the fact that the E109K variant is hydrolytically active.

However, *Exo1^EK/EK^* MEFs differ from *Exo1^+/+^* cells and behave similarly to *Exo1^null/null^* MEFs in the cellular responses to certain types of DNA damage (28). Thus, *Exo1^EK/EK^* cells display elevated frequencies of chromosome breaks and modestly enhanced resistance to N-methyl-N’-nitro-N-nitrosoguanidine. Although coupled action of the mismatch repair and damage signaling pathways has been implicated in the checkpoint and apoptotic responses to DNA methylator damage (44), the latter phenotype is indicative of a defect that is presumably distinct from conventional mismatch processing. Because Exo1-E109K was presumed to be catalytically defective, these phenotypes have been interpreted in terms of a requirement for Exo1 hydrolytic function in double-strand break repair and methylator damage response pathways (28). In view of our finding that Exo1-E109K is catalytically functional, we suggest that these phenotypes are due to structural consequences of the E109K mutation, which could for example directly or indirectly perturb Exo1 physical interaction with other components of these pathways.
